# Multiplexing Spheroid Volume, Resazurin and Acid Phosphatase Viability Assays for High-Throughput Screening of Tumour Spheroids and Stem Cell Neurospheres

**DOI:** 10.1371/journal.pone.0103817

**Published:** 2014-08-13

**Authors:** Delyan P. Ivanov, Terry L. Parker, David A. Walker, Cameron Alexander, Marianne B. Ashford, Paul R. Gellert, Martin C. Garnett

**Affiliations:** 1 School of Pharmacy, University of Nottingham, Nottingham, United Kingdom; 2 Medical School, Queens Medical Centre, University of Nottingham, Nottingham, United Kingdom; 3 Children's Brain Tumour Research Centre, Queens Medical Centre, University of Nottingham, Nottingham, United Kingdom; 4 AstraZeneca, Macclesfield, United Kingdom; Baylor College of Medicine, United States of America

## Abstract

Three-dimensional cell culture has many advantages over monolayer cultures, and spheroids have been hailed as the best current representation of small avascular tumours in vitro. However their adoption in regular screening programs has been hindered by uneven culture growth, poor reproducibility and lack of high-throughput analysis methods for 3D. The objective of this study was to develop a method for a quick and reliable anticancer drug screen in 3D for tumour and human foetal brain tissue in order to investigate drug effectiveness and selective cytotoxic effects. Commercially available ultra-low attachment 96-well round-bottom plates were employed to culture spheroids in a rapid, reproducible manner amenable to automation. A set of three mechanistically different methods for spheroid health assessment (Spheroid volume, metabolic activity and acid phosphatase enzyme activity) were validated against cell numbers in healthy and drug-treated spheroids. An automated open-source ImageJ macro was developed to enable high-throughput volume measurements. Although spheroid volume determination was superior to the other assays, multiplexing it with resazurin reduction and phosphatase activity produced a richer picture of spheroid condition. The ability to distinguish between effects on malignant and the proliferating component of normal brain was tested using etoposide on UW228-3 medulloblastoma cell line and human neural stem cells. At levels below 10 µM etoposide exhibited higher toxicity towards proliferating stem cells, whereas at concentrations above 10 µM the tumour spheroids were affected to a greater extent. The high-throughput assay procedures use ready-made plates, open-source software and are compatible with standard plate readers, therefore offering high predictive power with substantial savings in time and money.

## Introduction

Rising attrition rates of over 95% in drug discovery despite growing Research and Development budgets remain one of the biggest problems of the pharmaceutical industry [1]. This is especially true in the field of brain tumours where drugs need to circumvent a number of barriers to reach their target. The most common reasons for drug failure are lack of efficacy on one hand and safety risks on the other. Preclinical disease models of increased biorelevance are needed so that drug performance and toxicity in-vitro matches in-vivo behaviour [2]. Cancer drug discovery still relies largely on culturing tumour cell lines in two-dimensional monolayers to test the effects of therapeutics. This simple reductionist model offered by monolayers bears little resemblance to the in-vivo situation and the results obtained rarely coincide with the outcomes of clinical trials [3]. Our interest in improving drug delivery to the brain [4] has pointed the need for establishing superior preclinical models to characterise the safety and efficacy of cancer treatment.

Three-dimensional cell culture has been reported to match many aspects of the true behaviour of tumours [5]. Culturing cells in 3D accounts for the complex cell-cell, cell-extracellular matrix interactions, and the formation of nutrient and oxygen gradients which tumours exhibit in-vivo [6]. Methods of culturing cells in 3D [7], [8] include polarised cultures using transwell inserts, multicellular spheroids, bioreactors, matrix embedded cells, scaffold based systems, hollow-fibre bioreactors and organotypic slices. Multicellular tumour spheroids can be cultured in a high-throughput format and offer the closest representation of small avascular tumours in-vitro [9], [10]. They possess the necessary cell and matrix interactions, exhibit nutrient and oxygen gradients, and express genes similar to the ones expressed by tumours in-vivo [11], [12]. Spheroids can be formed using a number of methods: spontaneous aggregation, bioreactors, spinner flasks, hanging-drop, liquid overlay, matrix embedding, polymeric scaffolds and microfluidic devices [13].

Although the advantages of using spheroids in cancer research have been known since the 1970s [14] monolayer cultures are still the primary form of cell based screening. That is because three-dimensional cultures have been notorious for their slow growth, expensive maintenance and the difficulties associated with viability determination in 3D. In order to match the ease and convenience of 2D assays the ideal 3D screen should be quick, reproducible and amenable to high-throughput using standard methods such as phase and fluorescent microscopy and standard plate readers. Two methods claim to have all of the above qualities and aim to replace monolayer cultures as the methods of choice for anticancer drug screens: hanging drop plates and overlay cultures. The hanging drop plates developed by InSphero [15] and 3D Biomatrix [16] utilise the 96 and 384 well format and rely on growing the spheroid in a hanging drop. Their main drawback is the need to transfer the spheroid to a normal 96 or a 384-well plate in order to probe viability and proliferation. The liquid overlay method overcomes these challenges and utilises either in-house prepared poly-hydroxyethyl methacrylate [17] and agarose [18] coated plates or commercially available ultra-low attachment plates [19]. Spheroids grown using the liquid overlay method are scaffold free and the extracellular matrix that keeps them together is naturally secreted by the cells [20]. Although this culture method can produce spheroids with diameters of 100 µm to over 1 mm the preferred size for analysis is 300–500 µm. This ensures that the right pathophysiological gradients of oxygen and nutrients are present along with a core of hypoxic quiescent cells thought to be responsible for the increased chemo- and radioresistance of spheroids and solid tumours [18], [21], [22]. With all requirements met, liquid overlay is the most suitable method to grow reproducible 3D cell cultures of uniform well-defined shape accessible for automated high-throughput screens and data mining.

The replacement of monolayers by 3D cell culture will require validated, cost-effective, high-throughput compatible methods to assay spheroid growth, viability and the effects of treatment. Over 50 years of spheroid research has shown that the growth of cells in three dimensions is only advantageous in a practical sense if analysis is rapid and reliable in high throughput and with standard equipment. Since liquid overlay cultures are stationary and produce a single spheroid in the middle of each well, tracking growth can be easily accomplished with phase-contrast light microscopy. Images of the spheroids in each well can be collected and analysed using specialised equipment like the Celigo cytometer [19] or commercial software programmes [18], [19], [23]. However the investment in new equipment or image editing software can be seen as a hindrance to the mainstream implementation of spheroid research. Therefore we chose to work with the open-source software ImageJ and developed an in-house automated macro for spheroid analysis to facilitate image analysis within the scientific community. Apart from volume, cell viability within the spheroid can be assessed using metabolic assays like the reduction of Resazurin [24] or measuring ATP [19]. These assays are convenient and quick however they have not been properly validated yet for use in 3D cultures. Friedrich et al [25] have validated and encouraged the use of the acid phosphatase assay to determine viability and claimed that metabolic assays may not be equally suited for the task.

This paper describes work aimed at developing a biorepresentative three-dimensional cytotoxicity screen for human tissues with conventional microplate assays. The therapeutic and neurotoxic potentials of the model drug etoposide for brain tumours were investigated using spheroid volume, metabolism and acid phosphatase activity. The brain tumour medulloblastoma cell line UW228-3 was chosen to represent the pharmacological target of treatment and human foetal brain tissue spheroids were selected to determine possible off-target effects on the developing brain.

## Materials and Methods

### 1. Materials

Dulbecco's Phosphate Buffered Saline (PBS), Dulbecco's Modified Eagle's Medium - high glucose (DMEM), Ham's nutrient mixture F12, L-Glutamine solution 200 mM, Penicillin/Streptomycin solution (10,000 units penicillin and 10 mg streptomycin/mL), Heparin, Sodium pyruvate, Trypsin 10× solution 4-nitrophenyl phosphate disodium salt hexahydrate and etoposide were obtained from Sigma-Aldrich (Dorset,UK).

Foetal Bovine Serum (FBS), N2 supplement, B27 supplement serum-free supplement, DMEM without phenol red, basic human Fibroblast Growth Factor (bFGF), human recombinant Epidermal Growth Factor (EGF), Accutase and 0.4% Trypan Blue Stain solution were supplied by Invitrogen (Paisley, UK). Resazurin was sourced from Acros Organics (Loughborough, UK)

Ultra low attachment (ULA) 96-well round bottom plates were obtained from Corning (Amsterdam, The Netherlands)

### 2. Cell lines and culture

All experiments were performed in standard cell culture conditions at 37°C and 5% CO_2_.

UW228-3 medulloblastoma cell line [26] was obtained from Prof. Silber (University of Washington, Seattle, USA) with the help of the Children's Brain Tumour Research Centre at the University of Nottingham. Tumour cells were routinely cultured for less than 20 passages in monolayer in media containing DMEM (41.5%), Ham's F12 (41.5%), L-Glutamine solution (1%), Sodium pyruvate (1%) and FCS (15%). Subculturing was performed using 0.025% Trypsin in Ca^2+^ and Mg^2+^ free PBS solution for 5 minutes.

Foetal human brain tissue was received from the Joint MRC/Wellcome Trust (grant # 099175/Z/12/Z, Ethics committee approval 08/H0906/21+5, Health Research authority NRES Committee North East - Newcastle & North Tyneside 1) Human Developmental Biology Resource (www.hdbr.org). The tissue was rinsed, mechanically dissociated into a single cell suspension and cultured in non-treated flasks to form stem cell enriched neurospheres [27]. The Neural stem cell (NSC) defined serum-free media was made using DMEM (47.5 ml), Ham's F12 (47.5 ml), B27 (2 ml), N2 (1 ml), L-Glutamine (200 mM, 1 ml), Penicillin/Streptomycin solution (1 ml), hEGF (20 µg/ml, 100 µl), bFGF (10 µg/ml, 100 µl), Heparin (5 mg/ml, 100 µl) for 100 ml. Neurospheres were subcultured for less than 15 passages. Briefly, when the neurospheres reached a diameter of 100–300 µm they were collected in a polystyrene centrifuge tube, rinsed with PBS, resuspended in Accutase (1 ml) and agitated for 5 minutes at 37°C followed by mechanical dissociation with a blue (1 ml) tip on a Gilson pipette. The suspension was diluted with fresh NSC media and centrifuged at 300 g for 5 minutes. The cell pellet was resuspended in Ca^2+^ and Mg^2+^ free PBS (200 µl) with a yellow (200 µl) tip on a Gilson pipette and the final single-cell suspension diluted to the desired concentration with NSC media.

### 3. Spheroid production

Ultra low attachment (ULA) 96-well round bottom plates are pre-coated with a hydrophilic polymer that prevents attachment and triggers the formation of a single spheroid per well. Using these plates, spheroids of different size were formed in NSC media with both cell types using single-cell suspensions with a constant volume of 200 µl and concentrations ranging from 250 to 200 000 cells per ml. The plates were centrifuged lightly at 100 g for 3 minutes after seeding to bring the cells closer together, minimize cell death and encourage the formation of a single spheroid [17], [19]. Old media was carefully exchanged with fresh (150 µl) on days 3 and 5, taking care not to disturb the spheroids, and spheroids were cultured for 7 days before final analysis.

### 4. Phase microscopy and image analysis

Images of all spheroids were taken daily for growth determination and on day 3, day 5 and day 7 in cytotoxicity experiments using an Olympus CKX41 microscope with a 10× objective and an attached Olympus E330 camera. The scale of images was determined using a calibration slide. Images were analysed using the open-source software ImageJ (Fiji package) and a macro was written to automate the process (**Macro S1**). The macro works on whole folders of images, converts them to black and white, and uses the Yen thresholding algorithm [28]. It proceeds to clean any artefacts from the image, fills holes in the spheroid, separates it from debris and determines the area, maximum and minimum Ferret diameter of the spheroid. The macro also saves a copy of the file of each analysed image with a blue outline of the spheroids it has detected and an additional file with the numerical measurements for the whole folder. Variation in the area determination between the algorithm and manual measurement was found to be less than 5%. Data from the macro was analysed in Excel and the measured area (S) of the 2D projection of the spheroids was used to calculate the radius (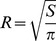) and the volume (V = 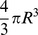) of an equivalent sphere [29].

### 5. Growth kinetics

UW228-3 cells were seeded in ULA plates at concentration ranging from 250 cells to 200 000 cells/ml and NSCs were seeded at 1000 to 200 000 cells/ml. They formed spheroids which were photographed daily and analysed for metabolic and acid phosphatase activity on day 7. Spheroid volume increase was calculated by dividing the difference in spheroid volume between day 7 and day 1 by the volume on day 1 (V_increase%_ = (V_day7_−V_day1_)*100/V_day1_).

### 6. Cytotoxicity experiments

Single cell suspensions were seeded in ULA plates at concentrations determined by the growth kinetics to produce spheroids between 300–500 µm in size on day 3 (25 k/ml for UW228-3 and 50 k/ml for NSCs). Old medium (150 µl) was carefully removed on day 3 and replaced with medium containing etoposide ranging from 0.03 µM to 300 µM from a 50 mM etoposide stock solution in DMSO. The drug exposure time was 48 h (until day 5) when medium was exchanged twice with fresh etoposide-free medium (150 µl), reducing drug concentrations to 1/16^th^ of initial levels. Afterwards spheroids were incubated for a further 48 h until day 7 when their viability was assessed using spheroid volume, resazurin metabolism and acid phosphatase activity. Negative control spheroids were cultured with 0.2% DMSO as vehicle and used to determine 100% viability while the positive control ones were exposed to 25% DMSO and represented 0% viability. The 300 µM etoposide concentration contained a higher level of DMSO (0.6%) and was used along with the positive control to elicit complete cell death and represent the bottom of the dose-response curve. A row of wells with media only and no cells was included to exclude contamination and verify that the positive control is functioning properly. Six replicate spheroids per condition were exposed to a total of 9 levels of etoposide in each experiment and the displayed results are the average of at least three independent experiments. In the case of neural stem cells, tissue from three different foetuses was used in the different experiments.

### 7. Resazurin reduction assay

A stock solution of resazurin (440 µM in PBS), was aliquotted and stored at −18°C. Frozen aliquots were thawed and kept in the fridge before use, protected from light. On the day of analysis a working solution of 60 µM resazurin was prepared in NSC medium. Medium in the wells was partially replaced with working solution (150 µl) and the plates were placed back in the incubator. Fluorescence was measured with an excitation wavelength of 530 nm and emission 590 nm on a Galaxy Fluostar plate reader at 4 h after dye addition.

### 8. Acid phosphatase assay

Acid phosphatase (APH) activity was determined using 4-nitrophenyl phosphate as described by Friedrich [18], [25]. The APH assay was performed on the same spheroids after the Resazurin assay. Resazurin was removed using two washes with PBS to leave 100 µl, APH assay buffer (100 µl), containing para-Nitrophenylphosphate (PNPP, 2 mg/ml), TritonX (0.1% vol/vol) in Citrate buffer (0.1M), was added and the plates incubated for 90 minutes at 37°C. Afterwards NaOH (1M, 10 µl,) was added to the wells and the absorbance was read at 405 nm with a reference wavelength of 630 nm on an Asys Expert 96-well plate reader.

### 9. Spheroid dissociation and cell counts

After volume and Resazurin assays, spheroids from the growth kinetics and cytotoxicity experiments were dissociated and counted. Dissociation was carried out after washing the spheroids twice with Ca^2+^ and Mg^2+^ free PBS (150 µl), removal of PBS, followed by 20 minute incubation with Accutase (50 µl) at 37°C. Mechanical dissociation with a multichannel pipette was carried out to form a single cell suspension and all six wells representing the same conditions were pooled in a microcentrifuge tube and centrifuged at 300 g for 5 minutes. The supernatant was taken off and the cells were resuspended in PBS (200 µl). Cell counts were performed using the Orflo Moxi Z automated thin-film sensor cell Coulter counter. The Moxi Z software has an internal curve-fitting algorithm which finds the healthy part of the cell population and expresses overall viability based on cell size reduction and debris content without the use of special reagents.

### 10. Assay Validation

Resazurin, Acid phosphatase and Volume determination assays were optimised and evaluated based on their Z-factor [30], Signal window [31] and Coefficient of Variation.

Z-factors were calculated using the equation:
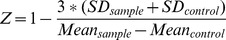
In growth experiments, the standard deviation and mean of the readings for medium-only wells were used as control. Z′-factors, reported in cytotoxicity assays, have been calculated by substituting the values for positive and negative control in the above equation. Signal window (SW) was defined as:
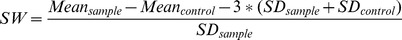
Coefficient of variation was calculated as 
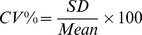


Acceptance criteria [32] were set at Z-factor>0.4, SW>2 and CV%<20%. They were used along with the biological considerations to optimise the cell density needed for the cytotoxicity screens. Plate uniformity was assessed on whole plates seeded with 25 k/ml UW228-3 and 50 k/ml NSCs cells. Phase contrast photographs were taken on day 3 after seeding and the variation in volume of the resultant spheroids was examined (acceptance criteria CV<20%). Signal variability validation was carried out on the 7^th^ day of the etoposide exposure experiments. Non-normalised assay readouts at each etoposide concentration were compared to the 25% DMSO positive control and Z-factor, SW and CV were calculated for each condition.

### 11. Data analysis

Results from volume, Resazurin reduction, APH activity and cell number measurements were analysed in MS Excel and Graphpad Prism 6. In assay validation experiments, readings for each assay were normalised so that the highest reading represents 100% and the reading for cell-free media 0%. Data was fitted to a straight line using Prism's least squares algorithm. In cytotoxicity experiments, readings were normalized so that untreated control has 100% viability and the readings for the positive control were taken as 0% viability. Dose response curves were fitted using either the four-parameter logistic equation for monophasic dose response (UW228-3) or the biphasic dose-response equation (NSCs) in Prism. Results are displayed as mean ± SD. Combined IC50 values from several experiments were derived by pooling the data together and analysing all runs from a single assay as one, using the logIC50 means (geometric means of IC50s) or by employing Prism's extra-sum-of-squares F-test to fit a curve with a common logIC50 between experimental runs as described in [33]. There were n = 6 replicates for each condition in each individual experiment and displayed data represent the mean of at least three independent experiments.

## Results and Discussion

Both neural stem cells and UW-228-3 tumour cell lines formed one centrally positioned spheroid in each well of the round bottom 96-well plates. Single spheroid formation and cell survival were encouraged with a light centrifugation which brought the cells together. Centrifugation reduced cell loss and yielded viable spheroids within 24 h with as few as 50 and up to 40000 cells. Centrifugation is reported to encourage paracrine signalling and suppress apoptosis in the early stages of spheroid formation [34]. The spheroids were cultured for 72 h before the first media change to allow for the formation of extracellular matrix and spheroid compaction. UW 228-3 medulloblastoma cells formed spheroids ranging from 92 µm (50 cells) to 840 µm (40×10^3^ cells) in diameter and coefficient of variation CV_diameter_ ≤5% (n = 6). The spheroids formed by NSCs were 150 µm (200 cells) to 730 µm (40×10^3^ cells) in diameter and CV_diameter_ ≤4% (n = 6). The culture in ULA plates was quick and reproducible and did not differ much from a regular monolayer screen except for the fact that the spheroids were left for 3 days before drug addition.

**Figure 1:**
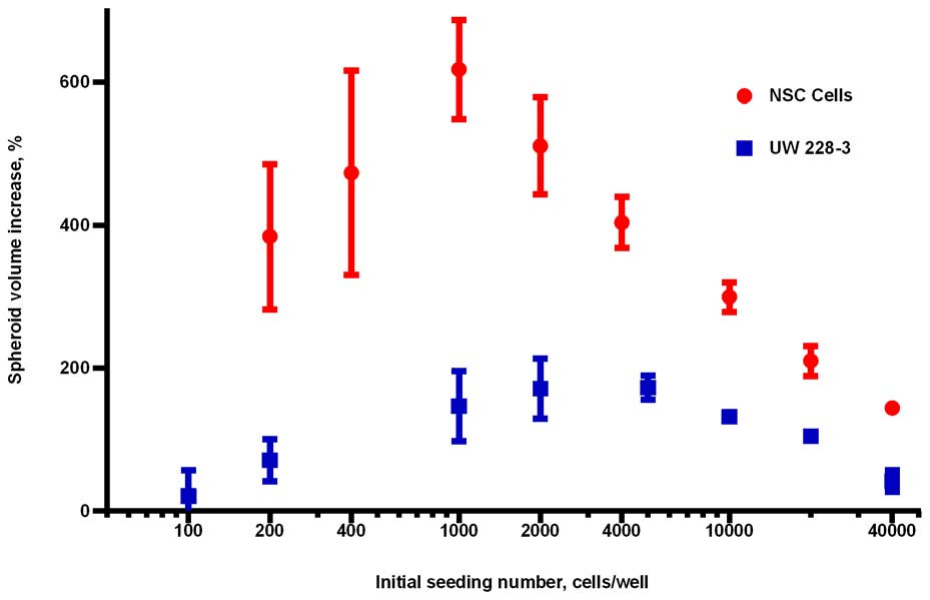
Spheroid volume increase of NSC and UW228-3 cells as a function of initial seeding number. Volume increase % = 100*(V_day7_−V_day1_)/V_day1_. NSCs grew more in a week than UW cells, reaching maximum growth increase of 600% for seeding 1000 cells per spheroid, whereas the maximum growth increase for tumour cells was around 170% for 2000–5000 cells/spheroid.

Stem cell and tumour spheroids exhibited different size increases over the 7 day duration of the experiment (**Figure 1**). Both cell types showed a similar relationship between seeding concentration and proliferation capacity. Very low seeding densities (50–100cells/well) resulted in little growth, intermediate ones (1000 and 5000 cells/well for NSCs and UWs respectively) proliferated the most, while seeding high cell numbers yielded big spheroids whose growth was hindered by the constant volume of media and the geometry of the well. Similar findings have been reported by Mori et al. [34], who argued that paracrine enhancement of Notch signalling in intermediate sized spheroids is one of the reasons for their enhanced growth. NSC media contains EGF and bFGF which stimulate the division of stem cells explaining their higher proliferation capacity compared to UW288-3. The decreased proliferation of the tumour cell line can be a consequence of having a lower percentage of stem-like cells responsive to EGF and FGF within the tumour spheroids and lack of interactions with normal tissue, which could enhance tumour growth [35]. Nevertheless, tumour spheroids increased their volume by 170% showing a slow and steady growth pattern close to their behaviour in-vivo.

**Figure 2:**
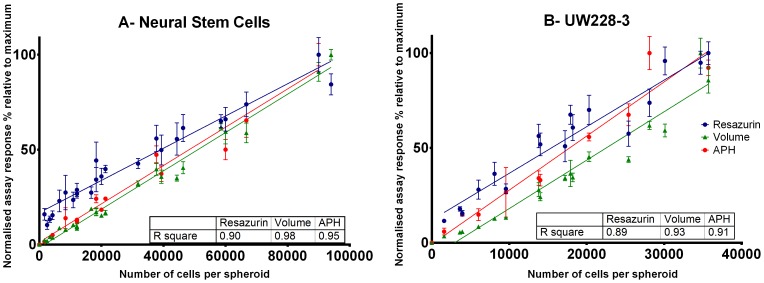
Volume, resazurin and acid phosphatase as methods to determine viability in NSC and UW228-3 spheroids. A. NSCs spheroids, diameter 200–800 µm. B. UW228-3 tumour spheroids, diameter of 160–700 µm. Both cell types were grown as spheroids for a week and then probed for volume, metabolic and acid phosphatase activity. Spheroids were enzymatically dissociated and normalised assay response plotted against the number of cells per spheroid in order to compare the three assays. Normalised assay responses from three independent experiments were pooled together, plotted and the linearity of each method was examined.

Apart from investigating growth patterns, these initial experiments were used to probe the suitability of spheroid volume, metabolic activity and acid phosphatase activity to predict numbers of viable cells within spheroids of various sizes of both cell types. Spheroids were grown for 7 days and their ability to reduce resazurin, acid phosphatase activity (performed on a second twin plate) and volume were determined as described above. Spheroids were dissociated and the resultant cell counts were plotted against assay response (**Figure 2**). The graphs clearly show that for healthy spheroids, over the range of 160–800 µm in diameter, volume correlates best with the number of healthy cells within a spheroid. As spheroids grow in size the cells in the core have less access to nutrients and oxygen, become firstly hypoxic and afterwards necrotic. Although the core of the spheroid becomes less populated the opposite is true for the periphery where a layer of densely packed cells is established [25], [36], [37]. This phenomenon can explain the relatively constant relationship between volume and cell number of the spheroids in this experiment. However this relationship will need to be confirmed and validated for every new cell type used and the relevant spheroid size as spheroids of >500 µm in diameter will have a more pronounced necrotic core and deviate from linearity [23], [38]. With the use of our specially written ImageJ macro (**Macro S1**) we were able to increase greatly the speed of image processing and facilitate the use of spheroid volume in rapid automated screens. The algorithm estimates spheroid volume using the area of the spheroid and fits the equivalent radius to that of an equivalent sphere. The spheroids do not need to be perfect spheres as the estimation is roughly valid for ellipsoids of width/length ratio up to 1.5 [29]. Moreover initial studies utilising the maximum and minimum Ferret diameter and estimating the volume of an ellipsoid (data not shown) exhibited greater variation due to thresholding artefacts affecting automatic measurements. The macro is optimised for phase-contrast images and requires manual magnification calibration at line 6. However the code can be easily adapted to suit applications like fluorescence imaging by altering the thresholding mechanism and using additional macros distributed with the free Fiji version of ImageJ [39], [40].

Acid phosphatase activity correlated almost linearly with cell number and volume for UW228-3 and NSCs. As evident from **Figure 2A**, in healthy NSC cells volume and acid phosphatase can be used interchangeably as markers of viability. Moreover, the correlation coefficient was above 0.9 for spheroids of both cell types indicating an excellent linear relationship. Although the APH method is faster and easier than photographing and computing spheroid volume it requires lysing the cells and has to be the final assay in a high-content analysis program.

The reduction of Resazurin, also known as Alamar Blue, by metabolically active cells was the final method for viability determination. Resazurin reduction was proportional to the number of cells within NSC and UW spheroids. However this method had a higher variability than volume and APH activity and the r^2^ values for Resazurin were the lowest of the three methods tested. Nevertheless, the Resazurin assay has the advantages of being non-toxic to the cells at the concentrations and time of exposure, can be used many times on the same cells and can also be multiplexed with other assays. Our initial concern with using Resazurin was that it may only detect metabolically active cells and miss hypoxic quiescent cells in the core of the spheroid. Cells in the periphery of the spheroid have good access to oxygen and nutrients and are actively dividing. Therefore their metabolism is much more rapid than the cells in the core of the spheroid where ATP levels have dropped to the minimum and metabolism is much slower [41]–[43]. In this way smaller spheroids were expected to be more metabolically active and appear more ‘alive’ than bigger spheroids which have a significant quiescent population [25]. This effect was observed in the NSC population (**Figure 2A**) and led to minor overestimation of viability for smaller spheroids.

Apart from viability validation the growth studies were also used to select the seeding concentration for both cell types that resulted in spheroid diameter at day 3 of around 400–500 µm, namely 5000 and 10000 cells/well for UW228-3 and NSCs respectively. The size was chosen because it fits the requirements for gradients of oxygen, nutrients and proliferation rate that are essential for a biorelevant spheroid screen [18].

Additionally, Z-factor, Signal window and Coefficient of variation were compared for the assays in both cell types at each seeding cell density after 7 days of culture in order to determine their suitability for high throughput screening. Both the Z-factor and Signal window take into account the variability of empty (media-only) control wells as well as the sample wells and provide a useful benchmark for hit-detection fitness in high-throughput screening (HTS). The coefficient of variation provides information on assay variability and can uncover pipetting problems especially at low seeding densities.

**Figure 3:**
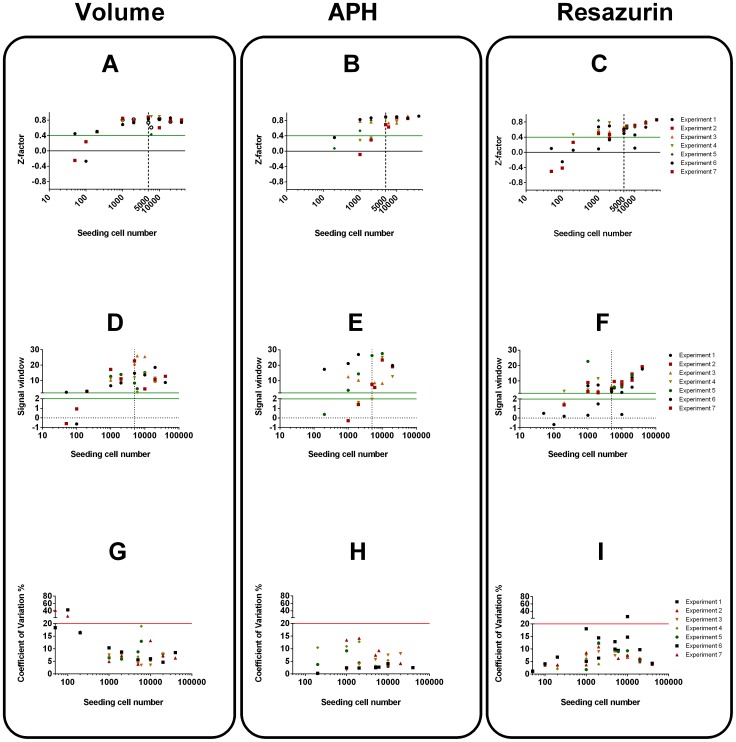
Assay characterisation using Z-factors, Signal Window (SW) and Coefficient of variation (CV) for UW228-3 cells. Z-factors (A–C), Signal window (D–F) and Coefficient of variation (G–I). Acceptance criteria Z-factor>0.4, SW>2 and CV<20% were colour coded so that values above the green lines meet quality criteria whereas values above the red line fail. Dotted line at 5000 cells represents chosen seeding density for spheroid cytotoxicity screening.

In UW228-3 cells **(** **Figure 3** **)** spheroid volume determination provided a sufficient working range for HTS when spheroids were seeded at density higher than 1000 cells/well. This high sensitivity is due to the ability of the thresholding macro algorithm to recognise empty wells and report them as such. Although the APH and Resazurin assays were also able to detect spheroids at the 1000cells/well, they excelled in all indicators at seeding concentration of more than 5000 UW228-3 cells/well. This along with the biorelevance arguments discussed above showed that seeding density of 5000 cells/well or more is optimal for cytotoxicity screening.

**Figure 4:**
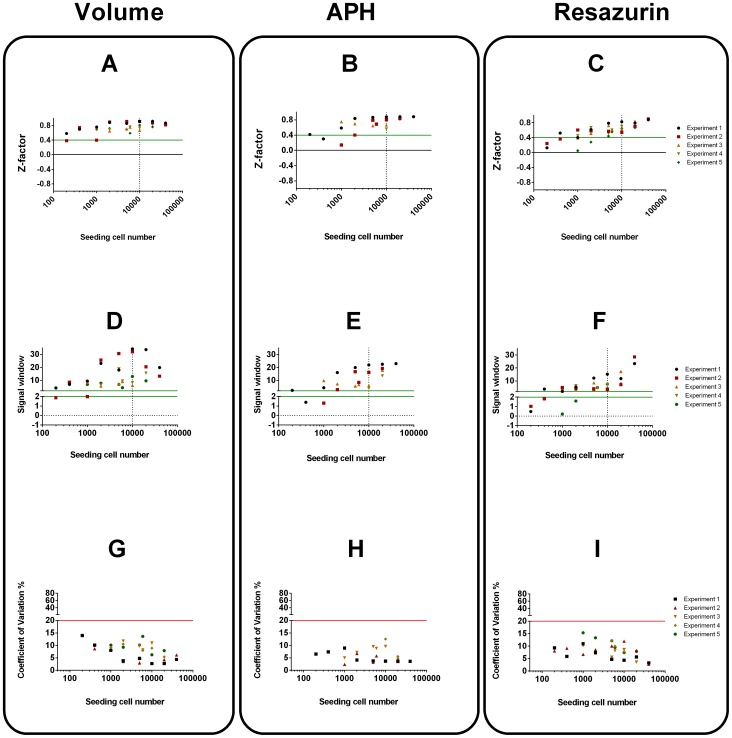
Assay characterisation using Z-factors, Signal Window (SW) and Coefficient of variation (CV) for NSCs. Z-factors (A–C), Signal window (D–F) and Coefficient of variation (G–I). Acceptance criteria Z-factor>0.4, SW>2 and CV<20% were colour coded so that values above the green lines meet quality criteria whereas values above the red line fail. Dotted line at 10000 cells represents chosen seeding density for spheroid cytotoxicity screening.

Neural stem cells produced spheroids with narrower size distribution and could be used in screens at even lower seeding densities **(** **Figure 4** **)**. Volume and APH had generally higher Z-factor and SW than Resazurin as their signals had lower variability. All parameters were within specification for spheroids initially made up of more than 2000 cells. Nevertheless a seeding density of 10000cells/well was chosen as it produced neurospheres of similar size to the tumour spheroids at the day of drug application.

The purpose of developing this screening assay was to compare the effects of etoposide on neural stem cells and tumours and to determine if it offers any selectivity in their action. The topoisomerase inhibitor etoposide [44] was picked as the drug of choice because it has shown promising activity against medulloblastoma in vivo [45] and has been investigated as a potential candidate for intrathecal therapy [4], [46]. The main therapeutic merit of etoposide is seen as a way of reducing craniospinal radiation in young medulloblastoma patients in whom it could reduce the serious side effects associated with radiotherapy [47].

Plate uniformity was assessed prior to etoposide addition at day 3. Spheroid uniformity was evaluated by the variability of spheroid diameter and volume along the whole plate in at least three plates on different dates **(Figure S1)**. The mean diameter for UW spheroids was 422 µm with a coefficient of variation of 4–7% between 7 plates of 33 to 66 spheroids each. NSC spheroids had a mean diameter of 463 µm and CV of 3% between 3 plates containing 66 spheroids each. The coefficient of variation for volume measurements was around 9% for NSC and ranged from 6 to 22% for UW228-3 cells with only one plate exceeding the 20% limit. Several outliers were identified and were attributed to deficiencies in pipetting technique and equipment. Therefore the Graphpad Prism ROUT method was used to eliminate outliers before testing for normality of volume distribution. The D'Agostino-Pearson omnibus K2 test showed a normal distribution of the cleaned volume data in all but one case. Even without outlier elimination a one-tailed t-test, for a sample of 6 replicates from the plate population, with α = 0.05 will have 1-β = 74% power to detect a 20% viability drop in UW228-3 cells (CV 15%) and 99% power to detect the same viability drop in NSC cells (CV 9%) [48].

**Figure 5:**
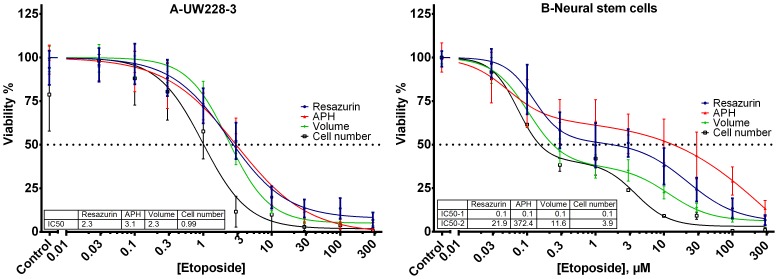
Dose-response curves for UW228-3 and NSCs spheroids exposed to increasing concentrations of etoposide. Normalized viability is expressed as volume, resazurin reduction, acid phosphatase activity and cell number. Data is pooled from at least three separate experiments.

**Figure 6:**
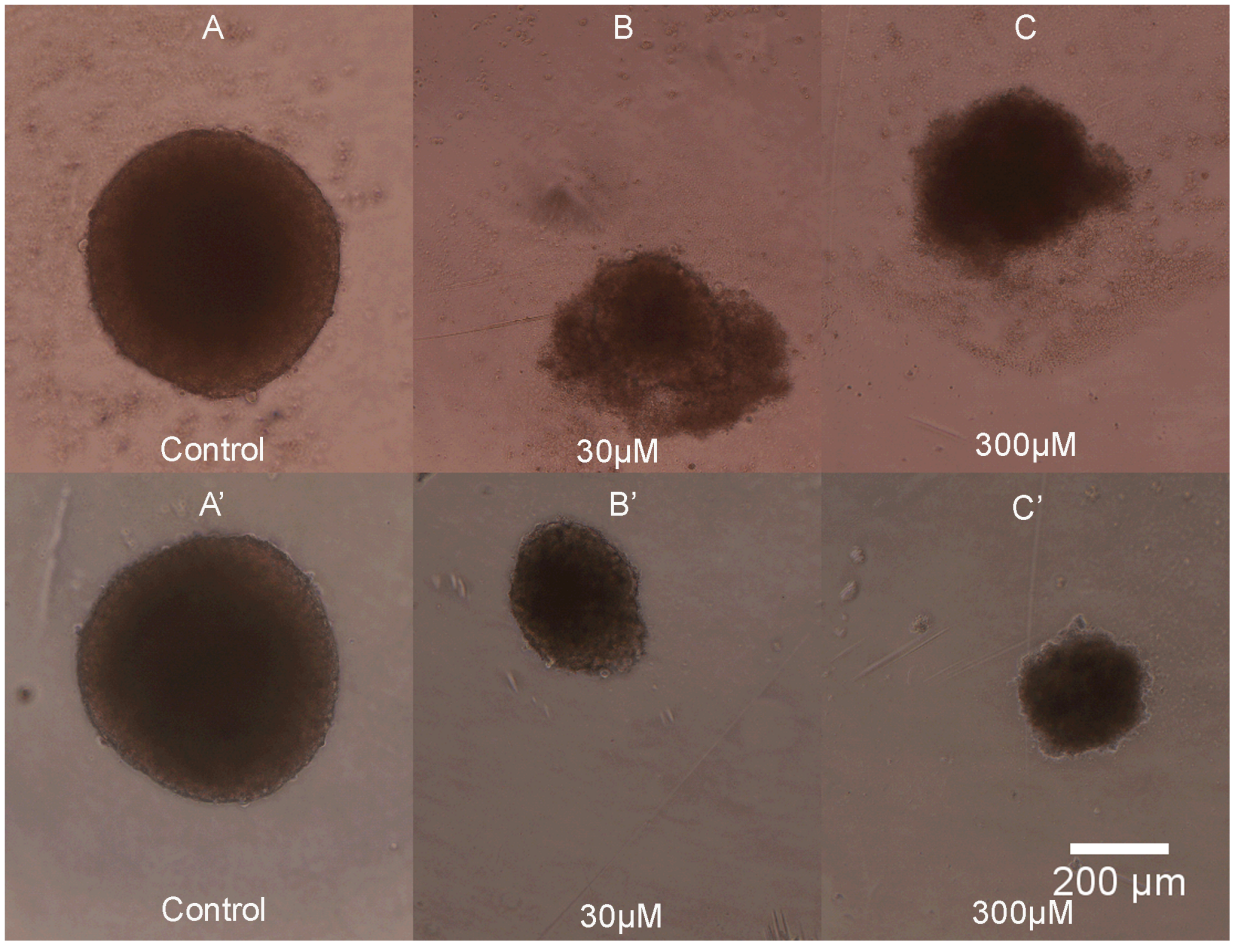
Phase-contrast microscope image of UW228-3 spheroids exposed to increasing concentrations of etoposide. Panels A–C show intact UW228-3 spheroids with a halo of debris and dead cells at high drug doses impeding image analysis. Panels A′–C′ capture the same UW228-3 spheroids after PBS rinse. Controls were cultured in plain media, concentration of etoposide is given in µM and scale bar applies for all images.

After the plate uniformity assessment, the tissues were exposed to etoposide for 48 h, followed by a 48 h period in plain media for the drug effects to fully manifest. The total duration time of the screen was 7 days and spheroid viability was determined using volume, acid phosphatase, metabolic activity and dissociated spheroid cell counts **(** **Figure 5** **)**. The dose-response curves for UW228-3 (**Figure 5A**) spheroids produced by reduction in volume (**Figure S2**), metabolism or acid phosphatase activity were very similar and the three assays appeared to be equally suited for a spheroid screen in this cell line. Viability determined by cell counts for dissociated spheroids was comparable to that calculated using the other assays up to drug concentrations affecting spheroid health. At pharmacologically active concentrations there appears to be an overestimation of cell death after subjecting the spheroids to enzymatic and mechanical dissociation. Apoptotic and stressed cells may be more sensitive to the dissociation process and that could be the reason behind the fast drop in viability estimated using cell numbers. Regarding phosphatase activity it is worth noting that at high drug concentrations the APH assay fails to detect any enzymatic activity in UW228-3 cells, whereas there was still some signal present from the Resazurin assay. Initially the volume measurements for the tumour cell line at high drug doses were thought to be less reliable because the spheroids were surrounded by a cloud of debris and dying cells and it was not possible to distinguish the dead cells from the living ones without bias **(** **Figure 6, panels A–C** **)**. Similar observations about the difficulties in volume measurements have also been reported by Friedrich [25]. However it was soon apparent that the debris and apoptotic cells can easily be washed out by exchanging the media twice with PBS **(** **Figure 6, panels A′–C′** **)**. This greatly facilitated automated image analysis by improving the speed and accuracy of spheroid size measurements.

Contrary to the UW228-3 monophasic response, foetal brain tissue-derived NSCs (**Figure 5B** **, Figure S3**) had a biphasic etoposide dose-response curve. Initially there was a very sharp decrease in viability down to 50% at concentrations approaching 0.3 µM. Beyond this concentration point the viable cell fraction decreased only slightly when etoposide concentrations were increased from 0.3 to 3 µM. This was followed by a moderate decrease in viability down to around 5% at the highest drug concentrations. The biphasic behaviour of the NSC spheroids is a sign that there are at least two distinct cell populations within the microtissues. The gradients of nutrients and oxygen can trigger differentiation into glia and neurons which would have a different sensitivity to the parent stem cells. Moreover, there could be an indigenous population of partially-differentiated progenitor cells in the foetal brain tissue which have a limited division potential and differ from the true stem cell phenotype [37], [49].

Viability estimates for NSC spheroids using the suite of four methods varied more than those for the UW228-3 cell line. That was probably due to the heterogeneous character of the tissue derived from foetal brains. Viability estimates using cell number and volume were of similar magnitude and were both generally lower compared to the values determined by resazurin and APH. Despite the fast drop in spheroid volume and cell counts, the metabolic activity as determined by resazurin reduction, dropped more slowly. The innate features of apoptosis, which starts with cell shrinkage while metabolic activity is not impaired, can give a possible explanation to these differences. Treatment with increasing concentrations of etoposide would push some of the cells in the spheroid towards apoptosis, leading to cell shrinkage and reduction in spheroid volume. It could also make the affected cells more sensitive to enzymatic digestion and the effects of mechanical agitation, leading to cell loss upon spheroid dissociation. However the apoptotic cells within intact spheroids would remain metabolically active, continue to reduce Resazurin and register as alive in the assay. Similarly to our findings, Chan et al [50] noted a difference in viability estimation between various cytotoxicity assays being developed for high throughput screening in 2-D assays. In some experiments using etoposide they showed that ATP and metabolism-based assays underestimated cytotoxicity compared to cell number. They have attributed this to increase in cell volume and mitochondrial mass relative to cell number. Other studies have also demonstrated increased ATP content and mitochondrial activity during etoposide treatment and have linked this with apoptosis [51], autophagy [52] or AMPK activation [53]. The viability measurements using acid phosphatase enzymatic activity against PNPP were the highest of all four assays. That was most pronounced for high etoposide concentrations between 10 and 100 µM where the fraction of apoptotic cells was the highest. Acid phosphatase is a digestive enzyme and has a role in cell death, apoptosis and autophagy [54]. The extensive cell kill induced at high etoposide concentrations could be triggering an increase of specific and non-specific phosphatase activity in stem cells. The biphasic curve also hints at the possibility that there are two cell populations with different drug sensitivity and enzymatic activity. The first population which is very sensitive to etoposide has a relatively low phosphatase expression and a more resistant second population which expresses higher APH activity.

**Figure 7:**
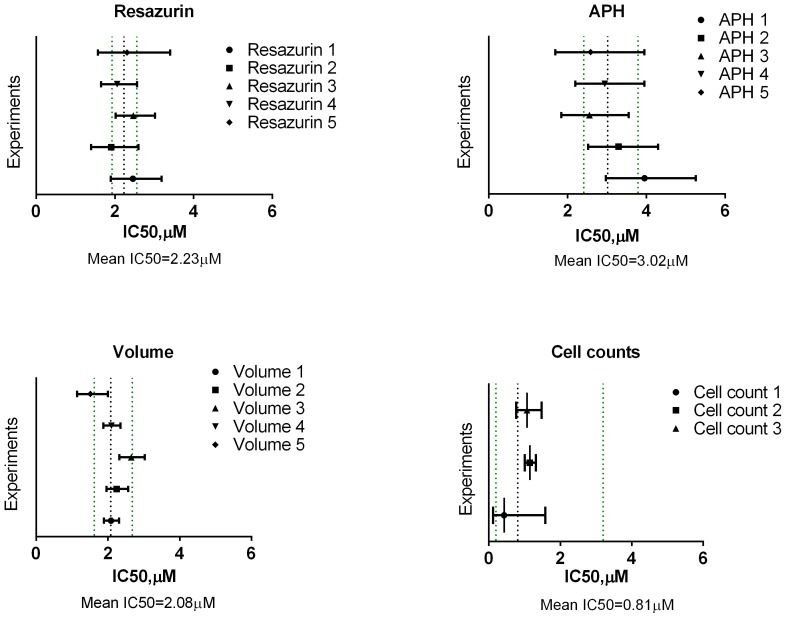
Confidence intervals for etoposide IC50 determinations for different assays in UW228-3 cells. The 95% confidence intervals (CI) for each experiment were plotted against the geometric mean (black dotted line) and 95% CIs (green dotted lines) for all individual experiments for each assay.

The precision of the four assays for UW228-3 cells (**Figure 7**) was assessed by comparing the 95% confidence intervals (CIs) for each experimental IC50 determination to the geometric mean values for all IC50 determinations along with the associated 95% confidence interval of the mean. The geometric mean of all experiments was calculated using the logIC50 values which have a distribution closer to normal as opposed to IC50 results which tend to be skewed [55]. This approach was chosen after comparing it to the methods of pooling the data into one or using Prism's extra-sum-of-squares F-test to compare IC50 values of dose-response curve fits [33] **(Figure S4**). It was deemed useful as a graphical aid to assess between-run variability and gave slightly broader CIs as seen in the case for Cell counting for example. Overall, resazurin and volume assays were superior to APH and direct cell counting. Although estimating viability using volume exhibited the smallest confidence intervals for the individual measurements, the IC50 values between runs varied more than those for resazurin. Moreover resazurin had the narrowest 95% confidence interval for the mean of the five separate runs.

**Figure 8:**
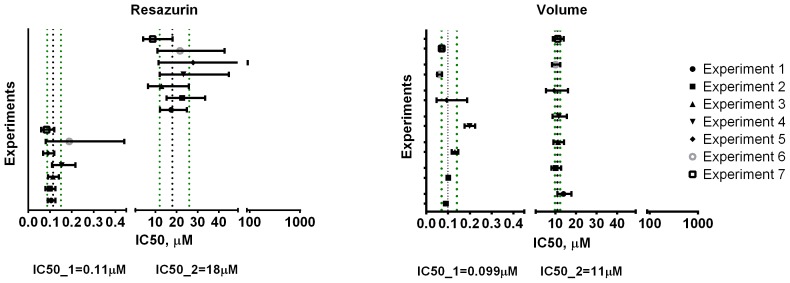
Confidence intervals for etoposide IC50 determinations for resazurin and Volume in neural stem cells. The 95% confidence intervals (CI) for each experiment were plotted against the geometric mean (black dotted line) and 95% CIs (green dotted lines) for all individual experiments for resazurin and Volume determinations.

For assay precision in neurospheres, only Resazurin and Volume gave IC50 values that were reproducible and had reasonable 95% confidence intervals varying less than one order of magnitude (**Figure 8**). Volume determinations yielded the tightest CIs with the highest level of precision out of the four assays. The determinations of IC50_1 and IC50_2 from APH and Cell counting varied over two orders of magnitude and were not included in the graph. The high level of variability in cell number estimation is due to the extra number of steps required to dissociate the spheroids and the possibility for cell loss during the process of mechanical and enzymatic cell separation. The APH assay, on the other hand, may have been affected by non-specific substrate cleavage at high etoposide concentrations leading to overestimation of viability and poor non-linear regression fits.

Additionally, signal uniformity assessment was performed on all etoposide treated plates to determine variability at each concentration. This test is similar to the signal variability assessment in the NCAT's Assay guidance manual [32] but instead of only using high, medium and low signal points we have used the whole dose-response curve to determine Z-factors (**Figure S5**) and Coefficient of Variation (**Figure S6**). The Z′-factors of all three assays were higher than 0.5 for the medium-only control wells and remained above the threshold of 0.4 even up to the IC50 concentration of 3 µM. This shows that the assays are well within their optimal working range for high-throughput screening at viabilities down to 50%. Although normalising the data did not affect the results of non-linear regression as described by Motulsky and Christopoulos [33], it was found to change the CV of the measurements and therefore CV calculations were done on the raw data before normalisation. CV was below 15% for most of the spheroids on the dose-response curve for APH and Resazurin assays. Volume had the lowest variability at low concentrations of etoposide, closely followed by the APH assay. However, the variability of volume measurements increased significantly in the wells where cell death was predominant (30–300 µM) making volume measurements less reliable at high etoposide concentrations despite the washing procedure. It is worth noting that despite the low CV% of the APH assay compared to Volume determinations and Resazurin, the precision of the APH IC50 fits was generally lower.

**Figure 9:**
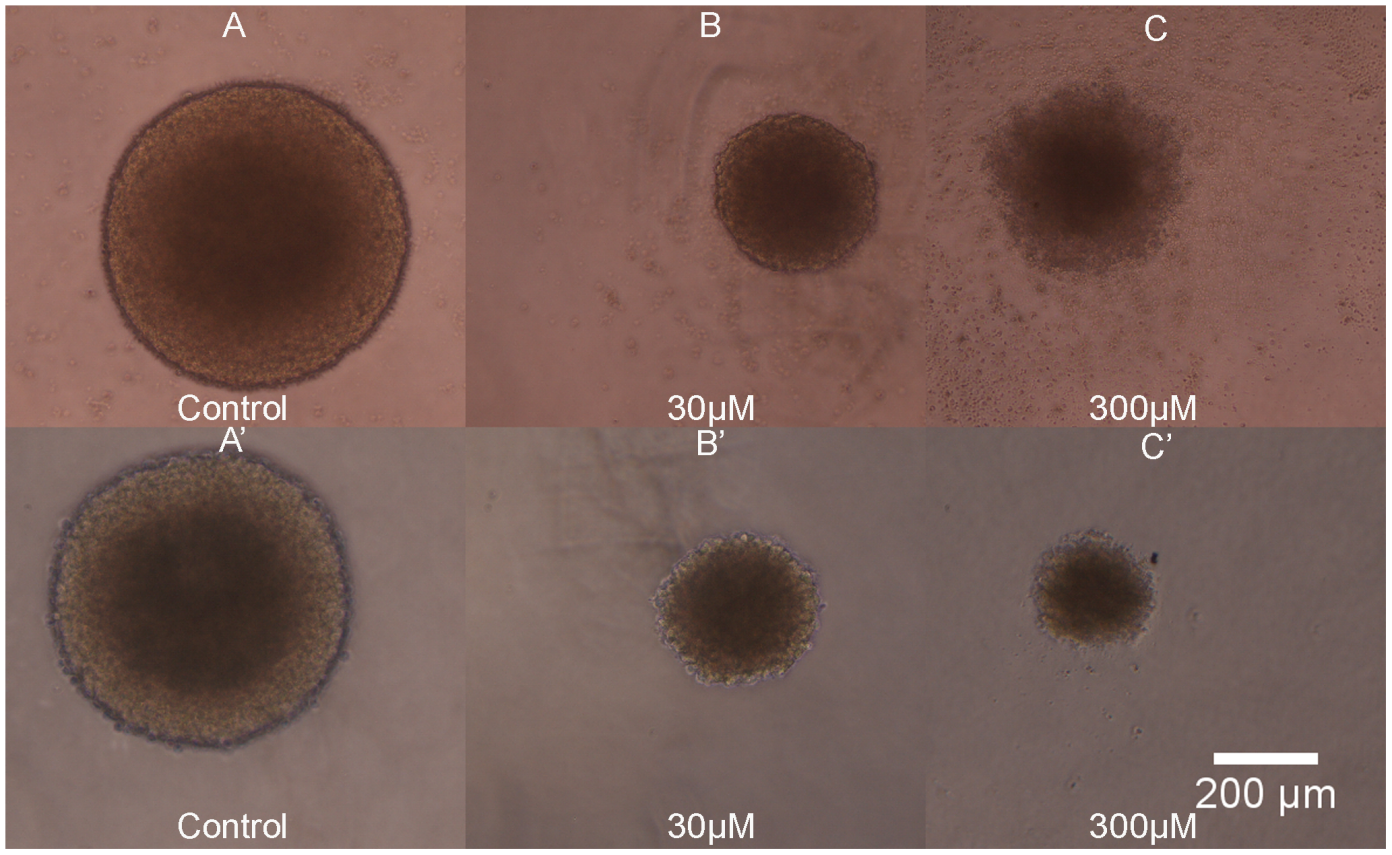
Phase-contrast microscope images of NSC exposed to increasing concentrations of etoposide. A–C spheroids before PBS wash. A′–C′- the same spheroids after PBS wash. Control is grown in plain media, concentrations of etoposide on drug treated spheroids shown in µM, scale bar applies to all panels.

Overall, volume measurements were the best method to study etoposide activity in foetal brain tissue closely followed by Resazurin reduction. Volume measurement sensitivity was greatly improved by washing off debris and dead cells with PBS similarly to the UW228-3 cells **(** **Figure 9** **)**.

Spheroid size reduction and metabolic activity determination complement each other as they use different mechanisms to estimate viability and can paint a fuller picture of spheroid health. When the rate of volume decrease is slower than the change in metabolic activity it would suggest that the proportion of dead cells, within the spheroid, is influencing the volume reading or that cells increase their volume due to treatment. However, a faster rate of volume decrease compared to resazurin reduction would indicate apoptosis-induced cell shrinkage without loss of metabolic activity. Indeed a proportion of larger cells with increased metabolic activity, as described by Chan et al [50] may be present in our neurospheres assay causing an underestimation of cytotoxicity in the case of volume and resazurin. Nevertheless viability estimates for volume and cell numbers were not statistically different for the most part of the dose-response curve. While some cells in the spheroids could increase in volume, others may shrink due to apoptosis and yet another group would detach from the spheroid bringing volume estimates for viability closer to cell numbers. Although live cell counts can be viewed as the “gold standard” for viability determinations in 2D, the extensive procedure for spheroid dissociation introduces variability outweighing the benefits of accuracy. Therefore, based on the lower variability of IC50 measurements and the similarities with actual cell numbers, in cases dealing with a new drug delivery strategy for a particular drug or with drugs with similar mode of action, volume would be a superior assay able to distinguish smaller differences in IC50s.

## Conclusions

Three-dimensional human cell culture is a useful tool that can help narrow the gap between preliminary in-vitro studies and in-vivo experiments that are required for drug development. Spheroids are cultured just as easily as monolayers in 96-well ultra-low attachment plates and a suite of assays can be employed to probe their viability. We have provided an open source ImageJ macro that automatically measures whole batches of spheroids and records the results both numerically and as an image. Spheroid volume was shown to be an excellent predictor for the number of viable cells in healthy spheroids. It can also be used as a reference method for cytotoxicity assays where the normalized volume readings are compared to other ways of estimating cell health. In this respect, the acid phosphatase assay was tested and its linear response to cell number in medulloblastoma spheroids of 160–700 µm validated. It is a simple, quick method for viability determination that does not require any expensive ingredients and is high-throughput compatible. However it relies on lysing the cells in question and needs to be the final assay in a high-content screening chain. The third assay tested, resazurin reduction, does not have these shortcomings because it is not toxic to the cells in the concentrations and exposure times used, it can be performed multiple times and coupled with other studies. The difference in metabolic rate between the cells in the periphery and the middle of the spheroid can account for the lower r squared values of resazurin data fit compared to the other two methods. Although it appears inferior to volume determination and APH, we have demonstrated that metabolic activity can reliably be used in cytotoxicity screens despite its perceived limitations. The optimal seeding densities for both cell types were determined by biological considerations for spheroid size and gradients and were also benchmarked for Z-factor>0.4, Signal window>2 and Coefficients of variation<20%. The suite of assays was performed on the same spheroids and the results compared and validated against the number of cells in a spheroid using both healthy tissue and spheroids exposed to a cytotoxic drug. Plate uniformity was examined for spheroid volume at day 3 and signal variability was assessed for volume, resazurin and APH assays during the cytotoxicity screen. After comparing the precision of IC50 determinations for all assays, cell volume and resazurin were found to perform better than APH and cell counts for both cell types. As volume, metabolism and acid phosphatase activity can all be influenced by cytotoxic drugs in a different manner, multiplexing those assays is the best way to get the true picture of cellular response. Since our interests lie in improving drug delivery in medulloblastoma we have only focused on one drug in this study. A useful future direction would be to explore a library of compounds which would allow for minimum significance ratio determination and better characterisation of the assays and cell response over an array of drugs with different mechanism of action [56].

Etoposide sensitivity of the UW228-3 medulloblastoma cell line was carried out in parallel with human foetal brain tissue derived stem cells (NSC) in order to have a comparison with cells representing human brain tissue. While the tumours exhibited a normal four parameter logistic dose-response curve, the NSCs had a biphasic response. The most likely explanation for this data is the presence of two sub-populations of cells within the neurospheres with a different sensitivity towards etoposide. The first sub-population had a low acid phosphatase activity and was more susceptible towards cytotoxic action, whereas the second one had a higher APH activity and was more resistant to topoisomerase inhibitors. The foetal NSC cells would be expected to have a relatively high proportion of stem cells. Under 3D culture conditions and the associated gradients of oxygen and nutrients the population of early progenitor cells can differentiate into late progenitors, neurons and glia which would have different rates of division and different sensitivity to etoposide. The faster dividing and more sensitive cell population is probably the less-differentiated one. Those undifferentiated cells are responsible for the growth, development and repair in vivo. While they make up a higher proportion of the brain in childhood when the brain is still growing and developing, they are confined to specific locations in the adult brain and have a supportive role. Establishing the proportions of stem-like, neural and glial cells that make up the neurospheres and how those change during etoposide exposure would bring greater insight into the off-target effects of topoisomerase II inhibitors. Furthermore the relative cell type proportions in the neurospheres could be influenced by changes in the media, such as EGF and FGF withdrawal that would promote progenitor differentiation into neurons and glia. Nevertheless, foetal brain tissue as a whole was more sensitive to etoposide up to concentrations of 5–10 µM when the neurospheres' slow decrease in viability was surpassed by the sharp decline in tumour cell survival. This is a biorelevant concentration that has been established to be tolerable in humans thereby inferring some limited selectivity of free etoposide [57].

Although etoposide is not generally regarded as a neurotoxic drug [58] there are reports which have demonstrated neurotoxicity in mice after blood-brain barrier disruption [59]–[61]. The heightened sensitivity of a sub-population of the neurosphere cells to etoposide can be explained by the presence of EGF and FGF in the culture media which limit differentiation and stimulate division. In the normal human brain *in-vivo*, only a small percentage of the available neural stem cells proliferate whereas the others are quiescent and may be spared from the effects of the cytotoxic drugs [62], [63]. This study suggests that free etoposide is not discriminating against actively dividing tumour and stem cells at concentrations below 10 µM. To enhance selectivity, better methods for drug delivery are needed to improve the effectiveness of medulloblastoma chemotherapy. Strategies to enhance the selectivity of etoposide could be using an etoposide-bearing drug-delivery system [64]–[66] that primarily targets tumour tissue or intrathecal therapy [4] to target leptomeningeal tumour tissue.

This convenient screening method can be implemented with standard equipment and reagents and can be used for screening new agents and drug delivery systems targeting CNS tumours. It offers the opportunity to compare the effect of drug upon the tumour and brain thereby comparing efficacy against toxicity, enhancing the bio-relevance to human tumours in clinical practice [10], [59], [67], [68]. The correlation with previously reported experimental and clinical studies [57], [60]–[63] and the practical convenience of this assay procedure suggest that it should be considered as a possible replacement for some animal testing experiments dealing with drug efficacy, particularly in brain tumour types relevant to childhood.

### Data Availability

Data is publicly available on Figshare with the DOI: http://dx.doi.org/10.6084/m9.figshare.1041615.

## Supporting Information

Figure S1 **Plate uniformity assessment for volume and diameter of spheroids before and after outlier removal.** NSC and UW populations are marked according to experiment number. All populations, with the exception of UW1, had a normal distribution according to the D'Agostino-Pearson omnibus K2 test after outlier elimination using Prism's ROUT algorithm.
(TIF)

Figure S2 **UW spheroids treated with etoposide.**
(TIF)

Figure S3 **NSC spheroids treated with etoposide.**
(TIF)

Figure S4 **Methods of combining different IC50 determinations between experiments for UW228-3 cells.** Data was subjected to an F-test to find a common curve that described all runs (Prism's F-test); The mean of logIC50 values was used in the geometric mean method and combining all normalised readings from different runs together was employed in the pooling method. Error bars are 95% Confidence intervals. The * in Volume F-testing means that the calculated IC50 values were statistically different between runs according to the extra-sum-of-squares F-test.
(TIF)

Figure S5 **Plate uniformity for cytotoxicity tests.** Z-factors for Volume- A and B; Acid phosphatase- C and D; and Resazurin- E and F.
(TIF)

Figure S6 **Coefficient of variation for different assays of etoposide treated plates.** A and B- Volume; C and D-APH; E and F-Resazurin.
(TIF)

Macro S1 **ImageJ macro automating size measurements for a folder of phase-contrast spheroid images.**
(DOCX)
