# Supplementary figures and images for: Multiplexing Spheroid Volume, Resazurin and Acid Phosphatase Viability Assays for High-Throughput Screening of Tumour Spheroids and Stem Cell Neurospheres
Source: PLoS One. 2014 Aug 13;9(8):e103817. doi: 10.1371/journal.pone.0103817 (PMC4131917; doi:10.1371/journal.pone.0103817)

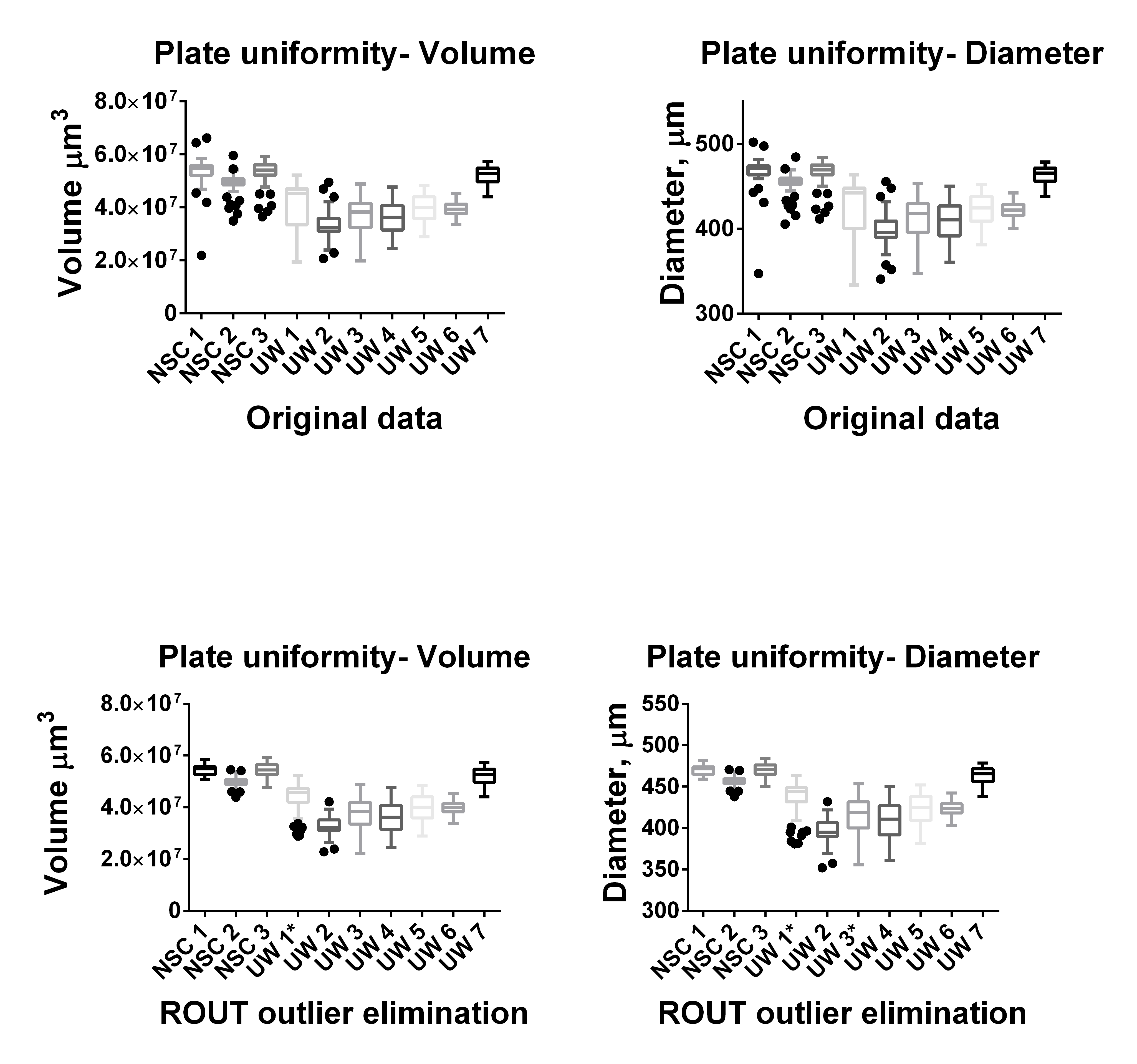

Supplement: Figure S1 — Plate uniformity assessment for volume and diameter of spheroids before and after outlier removal. NSC and UW populations are marked according to experiment number. All populations, with the exception of UW1, had a normal distribution according to the D'Agostino-Pearson omnibus K2 test after outlier elimination using Prism's ROUT algorithm. (TIF) [file pone.0103817.s001.tif]

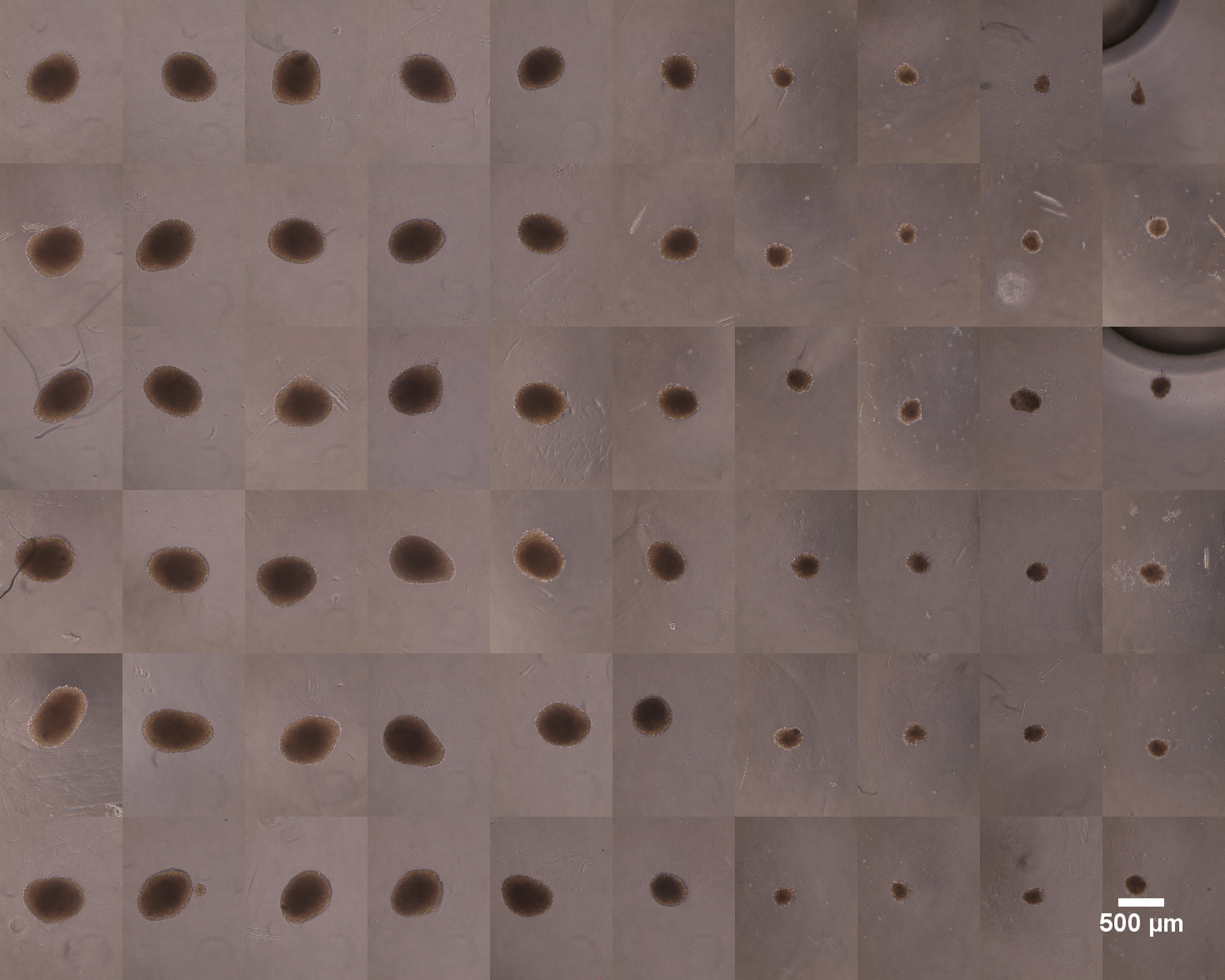

Supplement: Figure S2 — UW spheroids treated with etoposide. (TIF) [file pone.0103817.s002.tif]

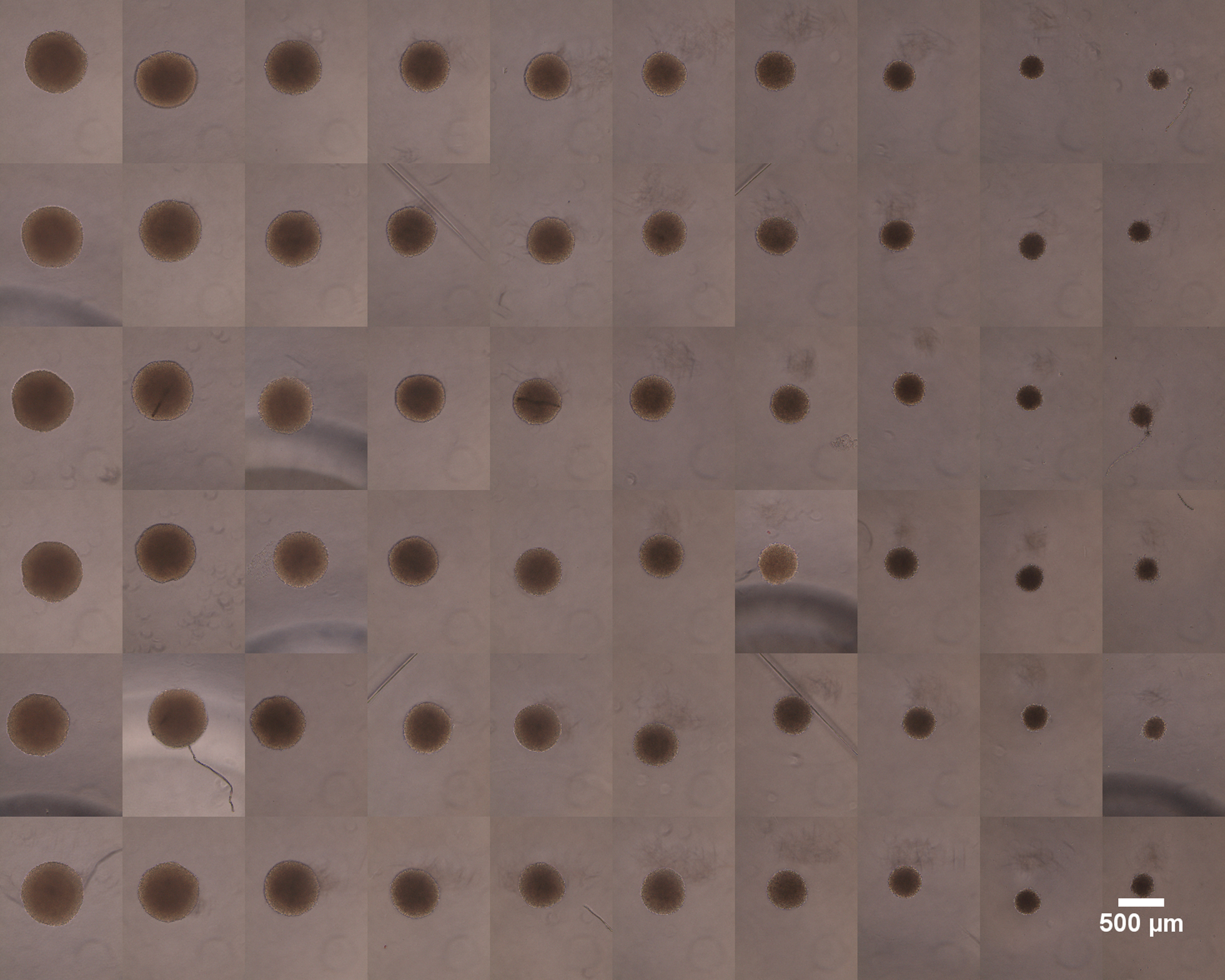

Supplement: Figure S3 — NSC spheroids treated with etoposide. (TIF) [file pone.0103817.s003.tif]

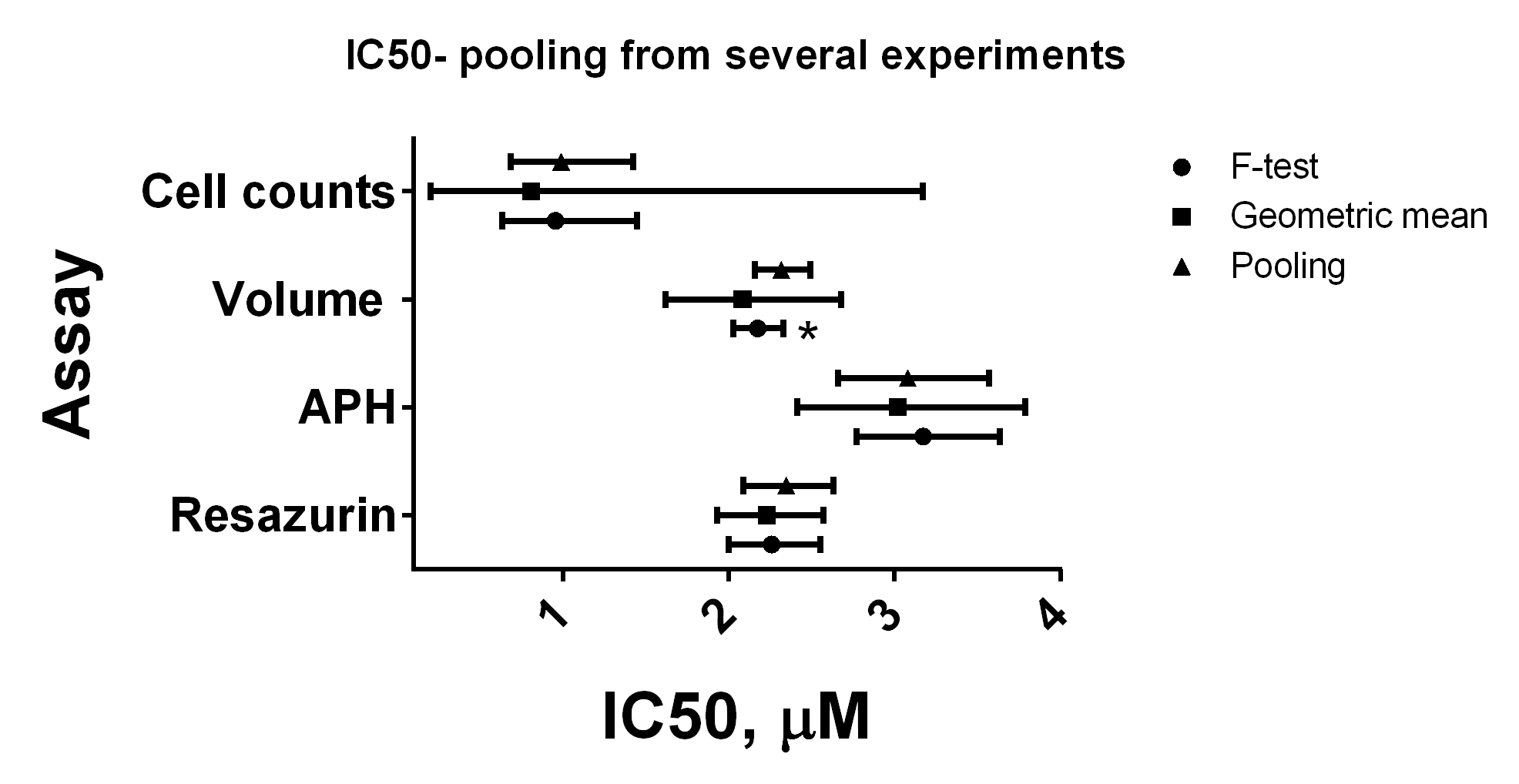

Supplement: Figure S4 — Methods of combining different IC50 determinations between experiments for UW228-3 cells. Data was subjected to an F-test to find a common curve that described all runs (Prism's F-test); The mean of logIC50 values was used in the geometric mean method and combining all normalised readings from different runs together was employed in the pooling method. Error bars are 95% Confidence intervals. The * in Volume F-testing means that the calculated IC50 values were statistically different between runs according to the extra-sum-of-squares F-test. (TIF) [file pone.0103817.s004.tif]

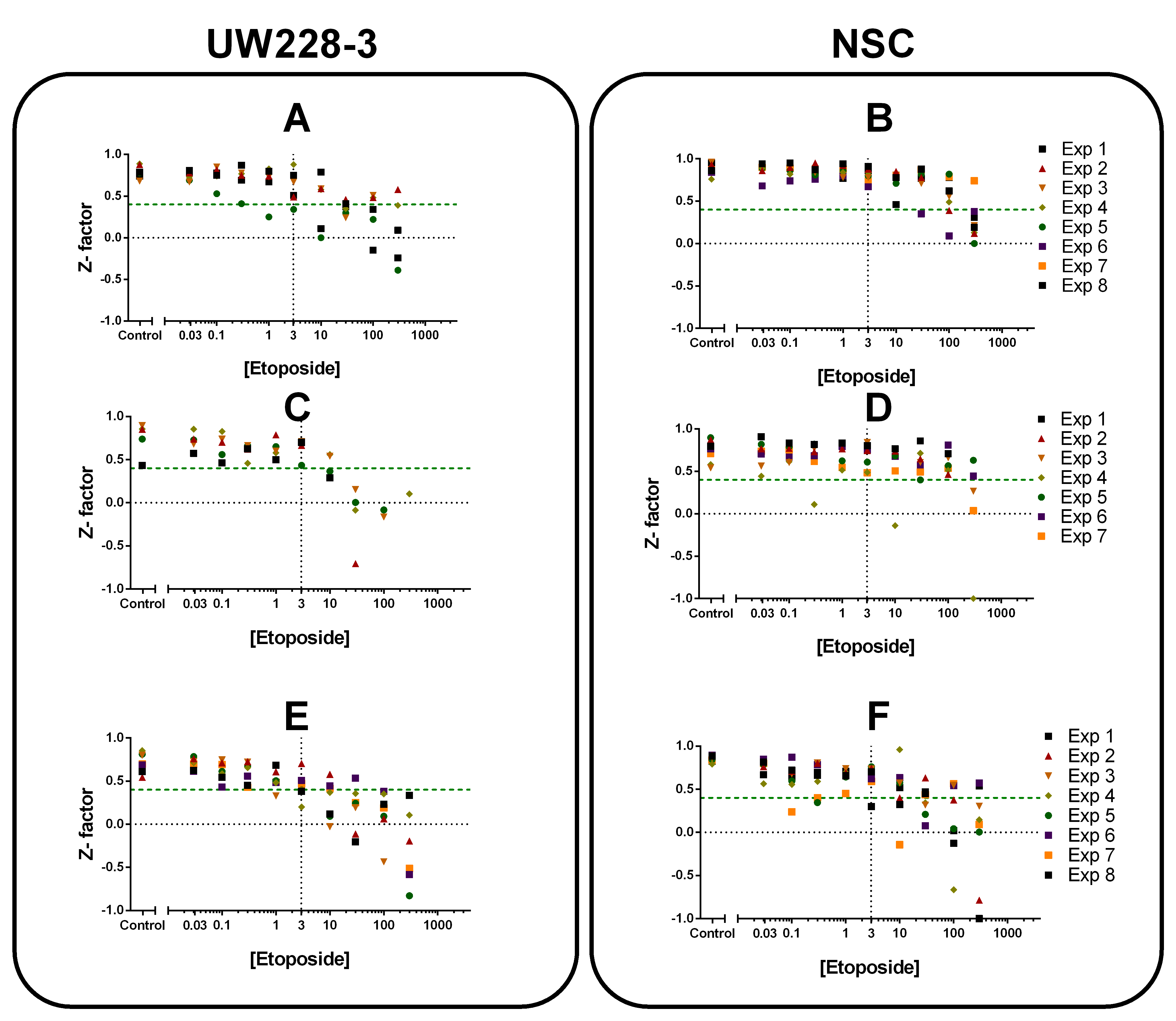

Supplement: Figure S5 — Plate uniformity for cytotoxicity tests. Z-factors for Volume- A and B; Acid phosphatase- C and D; and Resazurin- E and F. (TIF) [file pone.0103817.s005.tif]

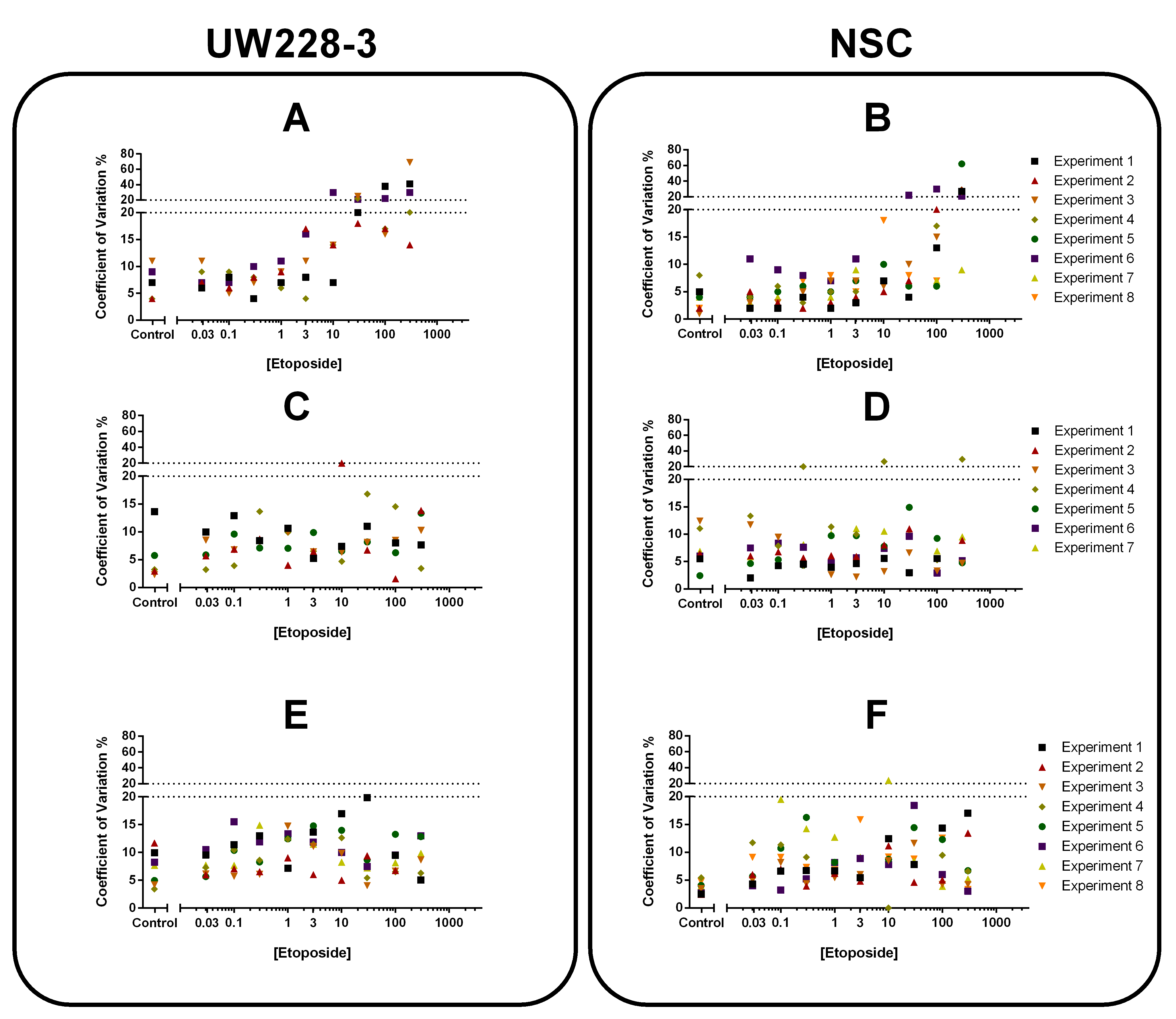

Supplement: Figure S6 — Coefficient of variation for different assays of etoposide treated plates. A and B- Volume; C and D-APH; E and F-Resazurin. (TIF) [file pone.0103817.s006.tif]
