# Supplementary material for: Multiplexing Spheroid Volume, Resazurin and Acid Phosphatase Viability Assays for High-Throughput Screening of Tumour Spheroids and Stem Cell Neurospheres
Source: PLoS One. 2014 Aug 13;9(8):e103817. doi: 10.1371/journal.pone.0103817 (PMC4131917; doi:10.1371/journal.pone.0103817)
Supplement: Macro S1 — ImageJ macro automating size measurements for a folder of phase-contrast spheroid images. (DOCX) [file pone.0103817.s007.docx]

//This macro aims to automate spheroid size measurement in three-dimensional cell culture. It requires input and output folders with images only, processes the images, records a file with spheroid measurements (Area, Ferret max, Ferret min, etc.) and writes an image with the outline/s of the determined spheroid/s.

//The spheroid detection and size determination function to be repeated for every image is defined below

function action(inputFolder,outputFolder,filename) {

open(inputFolder + filename);

//sets scale to predetermined values from calibration slide

run("Set Scale...", "distance=178 known=100 pixel=1 unit=µm global");

run("16-bit");

//run("Brightness/Contrast...");

run("Enhance Contrast", "saturated=0.35");

//Uses Yen thresholding algorythm

setAutoThreshold("Yen");

setOption("BlackBackground", false);

run("Convert to Mask");

//Gets the ratio between black (spheroid) and white (background) pixels. If we assume a single spheroid, the ratio between black and white pixels would allow us to estimate the size of the spheroid.

getHistogram(0,hist,256);

ratio = hist[255]/hist[0];

//If there are more pixels detected as spheroid(black) than background(white) then the spheroid has not been detected due to variations in background

if (ratio>1) {

// closes the image, reopens it, subtracts the background and proceeds as normal

close();

open(inputFolder + filename);

run("16-bit");

// Subtract Background is not used in the default function because it can lead to merging of spheroids and debris or it can remove the core of the spheroid leaving a very thin interrupted edge. In certain cases where the edges of a spheroid are very bright removing the background can give better results.

run("Subtract Background...", "rolling=50 light");

setAutoThreshold("Yen");

setOption("BlackBackground", false);

run("Convert to Mask");

run("Remove Outliers...", "radius=15 threshold=0 which=Dark");

getHistogram(0,hist,256);

ratio = hist[255]/hist[0];};

//The strategy here is to act differently according to spheroid size. The general pattern is to expand and then shrink back the spheroids in order to include all cells on the edges. Then a series of functions are used to remove noise and the Watershed function separates fused or superimposed particles. The Analyze particles function is targeted to the specific spheroid size according to the black/white pixel ratio.

if (ratio<0.001) {

run("Maximum...", "radius=8");

run("Fill Holes");

run("Minimum...", "radius=8");

//small spheroids require a more "gentle" function to clean up noise

run("Median...", "radius=2");

run("Maximum...", "radius=25");

run("Minimum...", "radius=25");

run("Fill Holes");

run("Watershed");

run("Analyze Particles...", "size=4000-Infinity circularity=0.20-1.00 show=[Overlay Outlines] display exclude include summarize");};

if (ratio >=0.001 && ratio<0.01) {

run("Maximum...", "radius=8");

run("Fill Holes");

run("Minimum...", "radius=8");

//slightly bigger spheroids and a more rigorous function to remove noise

run("Remove Outliers...", "radius=10 threshold=0 which=Dark");

run("Watershed");

run("Analyze Particles...", "size=10000-Infinity circularity=0.20-1.00 show=[Overlay Outlines] display exclude include summarize");};

if (ratio>=0.01 && ratio<0.2) {

run("Maximum...", "radius=8");

run("Fill Holes");

run("Minimum...", "radius=8");

run("Remove Outliers...", "radius=15 threshold=0 which=Dark");

run("Median...", "radius=4");

run("Watershed");

run("Analyze Particles...", "size=20000-Infinity circularity=0.20-1.00 show=[Overlay Outlines] display exclude include summarize");};

if (ratio>=0.2 && ratio<1) {

//Very big spheroids generally do not need to be expanded much to fill up the edges.

run("Maximum...", "radius=3");

run("Fill Holes");

run("Minimum...", "radius=3");

//Outliers and noise are removed rigorously

run("Remove Outliers...", "radius=50 threshold=0 which=Dark");

run("Minimum...", "radius=30");

run("Maximum...", "radius=30");

run("Watershed");

run("Analyze Particles...", "size=50000-Infinity circularity=0.20-1.00 show=[Overlay Outlines] display exclude include summarize");};

if (Overlay.size > 0) {

//Sends particles detected to the ROI manager

run("To ROI Manager");

close();

//Reopens the original image and pastes the outlines of the determined particles onto it

open(inputFolder + filename);

run("From ROI Manager");

outputPath = outputFolder + filename;

save(outputPath);

close(); }

else {

close();

};

call("java.lang.System.gc");

};

call("java.lang.System.gc");

run("Clear Results");

inputFolder = getDirectory("Choose the input folder!");

outputFolder = getDirectory("Choose the output folder!");

//Delete the next line if you want to see how the macro works on the images. However that will reduce processing speed.

setBatchMode(true);

images = getFileList(inputFolder);

//Sets the measurements that are recorded for each spheroid

run("Set Measurements...", "area centroid shape feret's display add redirect=None decimal=1");

//That is the cycle that runs through all images

for (i=0; i<images.length; i++) {

action(inputFolder,outputFolder,images[i]);

showProgress(i, images.length);

};

//Writes in the Results and Summary windows and saves the data.

selectWindow("Results");

saveAs("Measurements", "" + outputFolder + "Results.txt");

selectWindow("Summary");

saveAs("Text", "" + outputFolder +"Summary.txt");

setBatchMode(false);
